# Profiling non-coding RNA expression in cerebrospinal fluid of amyotrophic lateral sclerosis patients

**DOI:** 10.1080/07853890.2022.2138530

**Published:** 2022-10-31

**Authors:** Greig Joilin, Elizabeth Gray, Alexander G. Thompson, Kevin Talbot, P. Nigel Leigh, Sarah F. Newbury, Martin R. Turner, Majid Hafezparast

**Affiliations:** a School of Life Sciences, University of Sussex, Brighton, UK; b Nuffield Department of Clinical Neurosciences, University of Oxford, Oxford, UK; c Department of Neuroscience, Brighton and Sussex Medical School, University of Sussex, Brighton, UK; d Department of Clinical and Experimental Medicine, Brighton and Sussex Medical School, University of Sussex, Brighton, UK

**Keywords:** Amyotrophic lateral sclerosis, cerebrospinal fluid, biomarker, non-coding RNA, RNA-seq

## Abstract

**Introduction:**

Objective biomarkers for the fatal neurodegenerative disease amyotrophic lateral sclerosis or motor neuron disease (ALS/MND) are critical for diagnosis, drug development, clinical trials, and insight into disease pathology. Key candidates for biomarkers present in biofluids include non-coding RNA (ncRNA) transcripts including microRNA, piwi-interacting RNA and transfer RNA. To determine if the central nervous system was the source of the dysregulated ncRNA biomarkers we previously observed in serum, we sought to identify dysregulated ncRNA candidates in cerebrospinal fluid (CSF) which may provide new insight into the disease pathology.

**Methods and materials:**

Small RNA sequencing (RNA-seq) was undertaken on CSF samples from healthy controls (*n* = 18), disease mimics (*n* = 8), and ALS patients (*n* = 40) in our Oxford Study for Biomarkers of ALS cohort, with RT-qPCR used to confirm their dysregulation.

**Results:**

We found a range of ncRNA that were dysregulated in the RNA-seq screen, but these failed to be validated or detected in some cases using reverse transcription-quantitative polymerase chain reaction (RT-qPCR). Additionally, our previously identified serum ncRNA biomarker showed no change in CSF or correlation to serum.

**Conclusions:**

This study suggests the CSF may not be the source of dysregulated ncRNA in the serum and highlights the difficulty in identifying ncRNA in CSF as biomarkers for ALS.

**KEY MESSAGES**
- In this current study, we investigated the expression of non-coding RNA transcripts in the cerebrospinal fluid of ALS patients compared to healthy controls.
- RNA-seq identified dysregulated non-coding RNA transcripts, but these were not validated with RT-qPCR.
- We conclude that cerebrospinal fluid is not a suitable source of diagnostic biomarkers.

## Introduction

Motor neuron disease or amyotrophic lateral sclerosis (MND/ALS) is typically a fast-progressing fatal neurodegenerative condition with a lifetime risk of 1 in 300. It causes selective motor neuron loss, progressive muscle wasting, and ultimately death within two to five years after symptom onset, typically from ventilatory insufficiency [1]. Despite a common clinical core of combined upper and lower motor neuron signs, there is variation in the mix of clinical signs, the site of initial weakness, and the rate of disability progression. Further, while familial cases of ALS have highlighted genes with clear associations with the disease, including TAR DNA-binding protein 43 (*TARDBP*; TDP-43), chromosome 9 open reading frame 72 (*C9orf72*), and superoxide dismutase 1 (*SOD1*) [2], these account for less than 20% of all cases of ALS. As a result, diagnosis is a clinically evaluated disease exclusion process, which can result in diagnostic delay. Further, once a diagnosis is made, disease progression is measured through clinical evaluation which can vary and is limited in predicting prognosis. Thus, the identification of effective biomarkers will help to expedite diagnosis, allow for patient stratification, and assist in monitoring disease progression and treatment response.

The development of biomarkers that are minimally invasive to obtain, and relatively simple and quick to analyse is key for clinical use, and those derived from biofluids such as blood are ideal. In recent years, the short non-coding RNA (ncRNA) species, microRNA (miRNA) have been targets of interest because of their small size, resistance to degradation factors both internally (RNase) and externally (storage temperature, freeze/thaw cycles), and abundant expression [3,4]. As a result, a growing number of studies have investigated miRNA-based biomarkers in biofluids such as serum and cerebrospinal fluid (CSF) [5]. However, to date, very few biomarkers coincide across different studies, reflecting differences in methodology, sample preparation, and ethnic origin of the human samples. The main exception is miR-206, previously found to be up-regulated in muscle biopsies and in serum from ALS patients [6–9]. Few studies have investigated other ncRNA apart from miRNA as potential biomarkers in biofluids.

Recently, we investigated ncRNA expression in the serum of ALS patients across multiple cohorts using RNA-sequencing (RNA-seq) and identified seven ncRNA that were differentially expressed and have potential as diagnostic biomarkers [7]. The use of this unbiased transcriptome-wide approach allowed us to include small ncRNA such as piwi-interacting RNA (piRNA) and transfer RNA (tRNA) as potential biomarkers in addition to miRNAs. The resulting data identified two tRNA 5′ fragment and one piRNA alongside five miRNA that when modelled using a random forest model into a biomarker signature was able to correctly classify on average 74% of all samples as being from an ALS patient or disease mimics and healthy controls. This suggests that these ncRNA could be used as a useful tool for diagnosis.

The origins of these ncRNA however are unknown. Their presence in the blood could reflect cellular contents released from dying muscle caused by ALS or could be reflective of their role in the paracrine system of communication between cells [10,11]. They could also be reflective of changes occurring in the central nervous system (CNS) and the CSF, as it is known that extracellular vesicles, which can contain miRNA within them, are released by neural cells into the CSF [12,13] and can cross the blood brain barrier [14]. Indeed, it has been shown previously with RNA-seq that there are changes in miRNA expression in CSF samples from ALS patients [15]. As such, the dysregulation of ncRNA observed in the serum could reflect changes occurring in the central nervous system (CNS) and may provide new insight into the pathology of the disease. To explore this possibility, we utilised RNA-seq to identify dysregulated ncRNA in the CSF of ALS patients in comparison to healthy controls and disease mimics. Furthermore, we also profiled the seven ncRNA identified as biomarker candidates in the serum to see if they may have originated from the CSF. In addition to not confirming significant dysregulation of any candidates identified from the RNA-seq, none of the dysregulated ncRNA from serum were altered, suggesting an alternative source of these ncRNA.

## Methods

### Patient information

**Figure 1.**
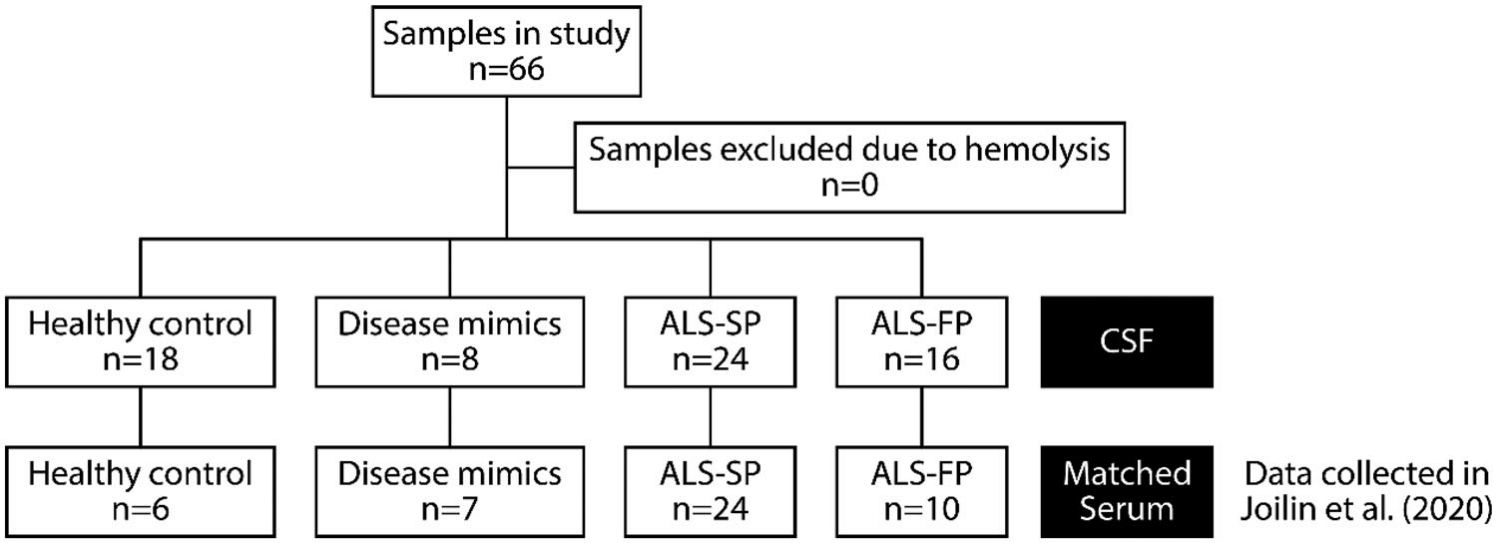
Flowchart of samples used in study.

Patients were recruited as part of the Oxford Study for Biomarkers in MND/ALS (BioMOx) from the Oxford MND Centre. Ethical approval for the collection and use of the biofluid samples and associated clinical data were obtained from South Central Oxford Ethics Committee B (08/H0605/85) and NRES Central Committee South Central – Berkshire (14/SC/0083). All participants provided written consent (or gave permission for a carer to sign on their behalf). The study included patients with ALS, patients with ALS mimic conditions (including multifocal motor neuropathy and Kennedy’s disease; henceforth referred to as disease mimics (DM)) and healthy control (HC) subjects (Table 1, Figure 1). Patients with ALS and mimic disorders were diagnosed according to standard criteria by two experienced neurologists (MRT, KT) and clinically re-assessed on the day of sampling (AGT, MRT). Healthy control subjects were typically spouses and friends.

**Table 1.** Characteristics of the sample cohorts whose serum was used in this study.

	HC	DM	ALS-SP	ALS-FP
Number of patients	18	8	24	16
Sex (M/F)	9/9	8/0	21/3	12/4
Age (year)	58.3	58.8	58.8	64.6
	(35 − 70)	(36 − 82)	(46 − 76)	(46 − 81)
Progression rate	–	–	0.32	1.25
			(0.05 − 0.6)	(0.6 − 5.1)
Mean disease duration (month)	–	–	30.8	13.0
			(5.3 − 88.5)	(2.37 − 25.5)
Onset (spinal/bulbar/both)	–	–	15/7/0	11/5/0
Genetic cases	–	–	C9orf72 (2)	C9orf72 (1)
			SOD1 (1)	

HC: healthy controls; NC: neurological controls; DM: disease mimics; ALS-SP: slow-progressing ALS patients; ALS-FP: fast-progressing ALS patients; M/F: male/female.

Clinical information collected included time from disease onset, diagnostic delay, mean disease duration, site of disease onset, presence of any ALS genetic markers, and clinical disability according to the ALS Functional Rating Scale-Revised (ALSFRS-R) scale. Patients were classified as having fast- or slow-progressing ALS based on their rate of change in the ALSFRS-R clinical rating (approximation of neurological status at disease onset minus ALSFRS-R score at the time of sampling divided by time from onset to sampling in months), with a threshold of 0.6 points per month selected based on the median value seen clinically to divide the samples.

### Sample collection, preparation, and RNA extraction

CSF samples were obtained by lumbar puncture directly into polypropylene collection tubes and centrifuged at 3000 rpm for 10 min at 4 °C within 1 h of sampling and stored at −80 °C until extraction. Total RNA was isolated from 400 µL of CSF samples using the miRNeasy Micro kit (Qiagen) with a DNase I treatment (Qiagen).

### RNA-sequencing

Healthy control, slow- and fast-progressing ALS (ALS-SP and ALS-FP respectively) samples were grouped into age- and sex-matched pools of four to five samples each and 7 µL of extracted RNA per sample were combined and concentrated to 8 µL using a SpeedVac at ambient temperature for 40 min. RNA-Seq libraries were then generated from the pooled RNA using the QIAseq miRNA library kit (Qiagen) following the recommended parameters for serum RNA. Libraries underwent 75 bp paired end sequencing on an Illumina NextSeq 500 machine at the University of Leeds, UK. Initial sequencing data were checked with FastQC (http://www.bioinformatics.babraham.ac.uk/projects/fastqc/) before reads were analysed on the Qiagen GeneGlobe platform (https://geneglobe.qiagen.com/) to remove 5′ and 3′ adaptors using cutadapt [16], with reads without UMI or less than 16 bp long removed. These data were sequentially aligned to a database of ncRNA transcripts using Bowtie [17] to GRCh38, and annotated according to mature miRbase transcripts, piRNA Bank transcripts, and features for tRNA, rRNA, mRNA, and other RNA. Read counts were then collapsed to the number of original transcripts using unique molecular indexes (UMI), and normalised and differential expression calculated between sample groups using DESeq2 [18].

### RT-qPCR confirmation and analysis

2 µL of RNA extracted from CSF was converted to cDNA using the TaqMan Advanced miRNA RT-qPCR chemistry following the manufacturer’s instructions. Candidate transcripts were profiled using pre-designed primers and master mixes (Applied Biosystems; miRNA commercially available probes; piRNA and tRNA: custom-designed probes; Supplementary Table 6) using fast cycling conditions on a QuantStudio 7 cycling machine. Quantitative cycle (Cq) values were averaged and normalised to the arithmetic average of hsa-miR-9-5p and hsa-miR-501-3p. An average for the healthy control samples was calculated and all samples were compared to this average for a ΔΔCq value.

### Statistical analysis

Statistical analysis for RT-qPCR were conducted on ΔΔCq values for each sample with GraphPad Prism 8.0. Outliers were identified using the ROUT method (*Q* = 1%). One-way ANOVA was carried out across the four groups with Tukey’s multiple comparison for parametric data and a Welch’s one-way ANOVA with Gomes-Howell multiple comparison when nonparametric according to a Shapiro-Wilks normality test. Correlations were analysed using Pearson’s rank correlation tests. Correlation analysis between matched serum and CSF samples utilised expression data for ncRNA expression that were collected as part of Joilin et al. [7]. All statistics were two-tailed and significance was set at *p* < 0.05.

## Results

### Diverse but limited number of ncRNA detected in CSF using RNA-sequencing

**Figure 2.**
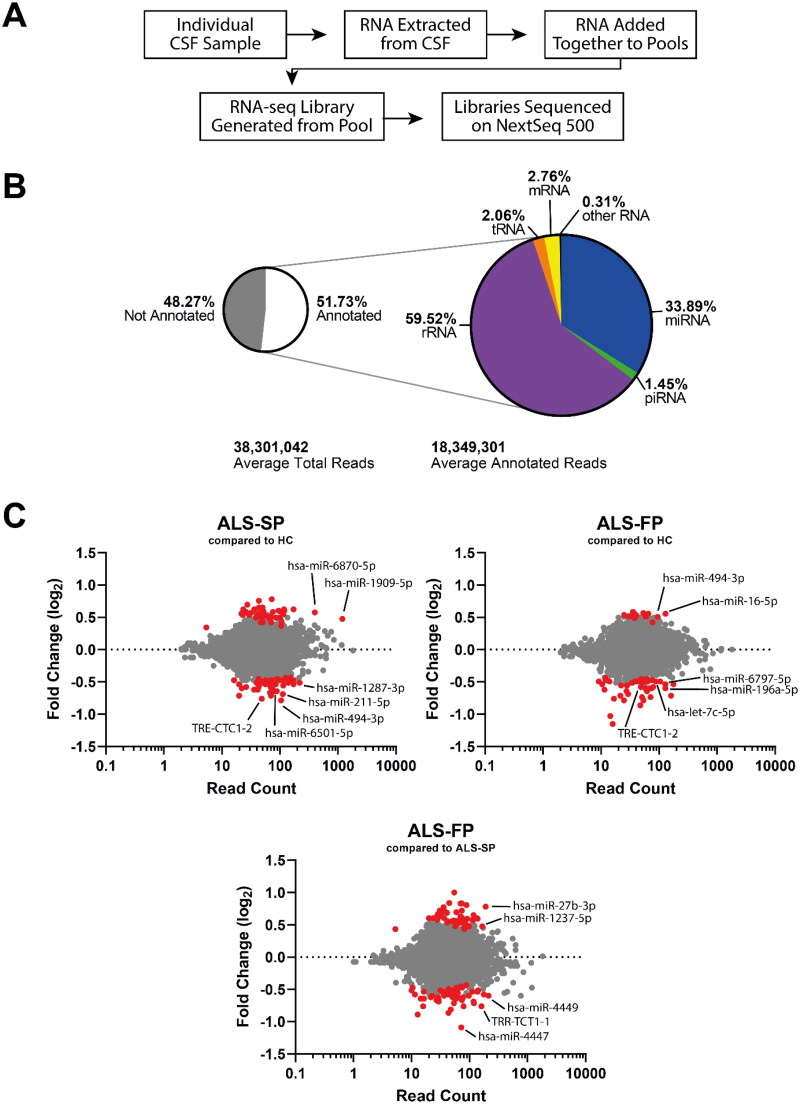
RNA-seq shows ncRNA may be dysregulated in CSF from ALS patients. (A) Workflow of the experiment from sample to sequencing data. (B) Summary of the number of aligned reads that were annotated and the ncRNA species they aligned to. (C) MA plots of ncRNA dysregulation in slow- (ALS-SP) and fast-progression (ALS-FP) ALS patients compared to healthy controls, and ALS-FP compared to ALS-SP. Dots in red were significantly dysregulated compared to healthy control (horizontal dotted line representing *p* < 0.05).

We sought to identify dysregulated ncRNAs in the CSF of ALS patients that could not only be used as potential biomarkers, but provide insight into the source of the ncRNA in serum and whether they were being trafficked between CSF and blood. To undertake an unbiased screen of dysregulated ncRNA, we undertook next generation RNA-seq to profile all small ncRNA in the CSF of healthy controls and ALS patients using our established protocols [7]. ALS patients were subdivided into slow- and fast-progressing (ALS-SP and ALS-FP respectively) based on the observed clinical median change in the ALSFRS-R score per month of 0.6 points. This included combining our samples into age- and sex-matched pools following RNA extraction but prior to library creation to increase the signal to noise ratio, which we had previously demonstrated identified dysregulated ncRNA in serum (Figure 2(A); Supplementary Table 1). Using the Illumina NextSeq, we were able to generate an average of 38 million reads per sample pool. All sample pools as expected showed comparable quality control statistics, with the 75 bp reads having on average sequence and base quality scores of Q31.

Using the analysis pipeline provided on the Qiagen GeneGlobe platform, removal of short and non-adapter reads resulted in 18 million reads per sample, of which we had an average alignment to the human genome (hg38) of 30.8%. Of those aligned, 51.73% were annotated (15.9% total reads post-processing). The majority of these reads were aligned to ribosomal RNA (rRNA) transcripts (59.5%) followed by miRNA (33.9%). The other forms of short ncRNA including tRNA and piRNA made up the remaining 6.6% (Figure 2(B); Supplementary Tables 2 and 3). Despite using increased amounts of CSF and maximum input of extracted RNA from these samples, it appears that there is little intact ncRNA in the samples, which concurs with the difficulties experienced in quantifying the RNA concentration in these samples. These data would suggest the small number of aligned reads and the high number of reads annotated to rRNA may be the result of low RNA input and diversity, supported by an inability to directly quantify RNA concentration using A260 or intercalating fluorescence dyes, and limited analysis of the RNA-seq (Supplementary Table 5). Importantly, there was very little PCR bias introduced, as shown by a high level of correlation (*r_p_* > 0.99) before and after correction for unique molecular indices (UMI) which are additional sequences that index each original individual transcript.

We next compared ncRNA expression in our slow- and fast-progressing ALS patients compared to our healthy controls (HC) in addition to between the two ALS groups. While the majority of transcripts did not show significant dysregulation, both groups did show both down- and up-regulation of ncRNA transcripts (Figure 2(C); Supplementary Table 4). Interestingly, ALS-FP had a skew towards down-regulation (46 ncRNA transcripts down-regulated vs 15 ncRNA transcripts up-regulated) in comparison to the more balanced dysregulation in the ALS-SP groups (down-regulated: 45; up-regulated: 43), and between the ALS-SP and ALS-FP groups (down-regulated: 60; up-regulated: 52). Of note, only four ncRNA were significantly dysregulated in both the fast- and slow-progressing ALS groups compared to healthy controls (TRE-CTC1-2, hsa-miR-494-3p, hsa-miR-885-5p, and hsa-miR-6501-5p).

### NcRNA not confirmed as dysregulated in CSF using RT-qPCR

We next sought to confirm the dysregulation of candidate ncRNA in the CSF of both slow- and fast-progressing ALS samples. Using the TaqMan Advanced miRNA assay chemistry, we first aimed to identify potential normalisers that showed consistent and correlated expression using NormFinder [19] on our RNA-seq data. This identified hsa-let-7a-5p, hsa-let-7b-5p, hsa-miR-9-5p, hsa-miR-30a, hsa-miR-204-5p, hsa-miR-501-3p, and hsa-miR-606. Of these, the two candidates that we verified as showing stable expression were hsa-miR-9-5p and hsa-miR-501-3p with an average Cq value of 30.3, a coefficient of variations of 5.2% and 3.7% across all samples respectively, with their average 4.1%, and their expression correlated with each other (*r_p_* (64) = 0.66, *p* < 0.0001).

A large number of ncRNA that showed large changes in the RNA-seq analysis also had low read counts across all sample pools, suggesting low expression that would be difficult to profile and have limited utility as biomarkers. Therefore, we limited our selection of candidate ncRNA to those that show significant dysregulation and sufficient expression levels (at least 50 reads on average across all pools) for further confirmation. These ncRNA included hsa-let-7c-5p, hsa-miR-9-3p, hsa-miR-196a-5p, hsa-miR-211-5p, hsa-miR-451a, and hsa-miR-6797-5p in our samples. Additionally, we also profiled the four ncRNA that were dysregulated in both the slow- and fast-progressing ALS patients (TRE-CTC1-2, hsa-miR-494-3p, hsa-miR-885-5p, hsa-miR-6501-5p).

**Figure 3.**
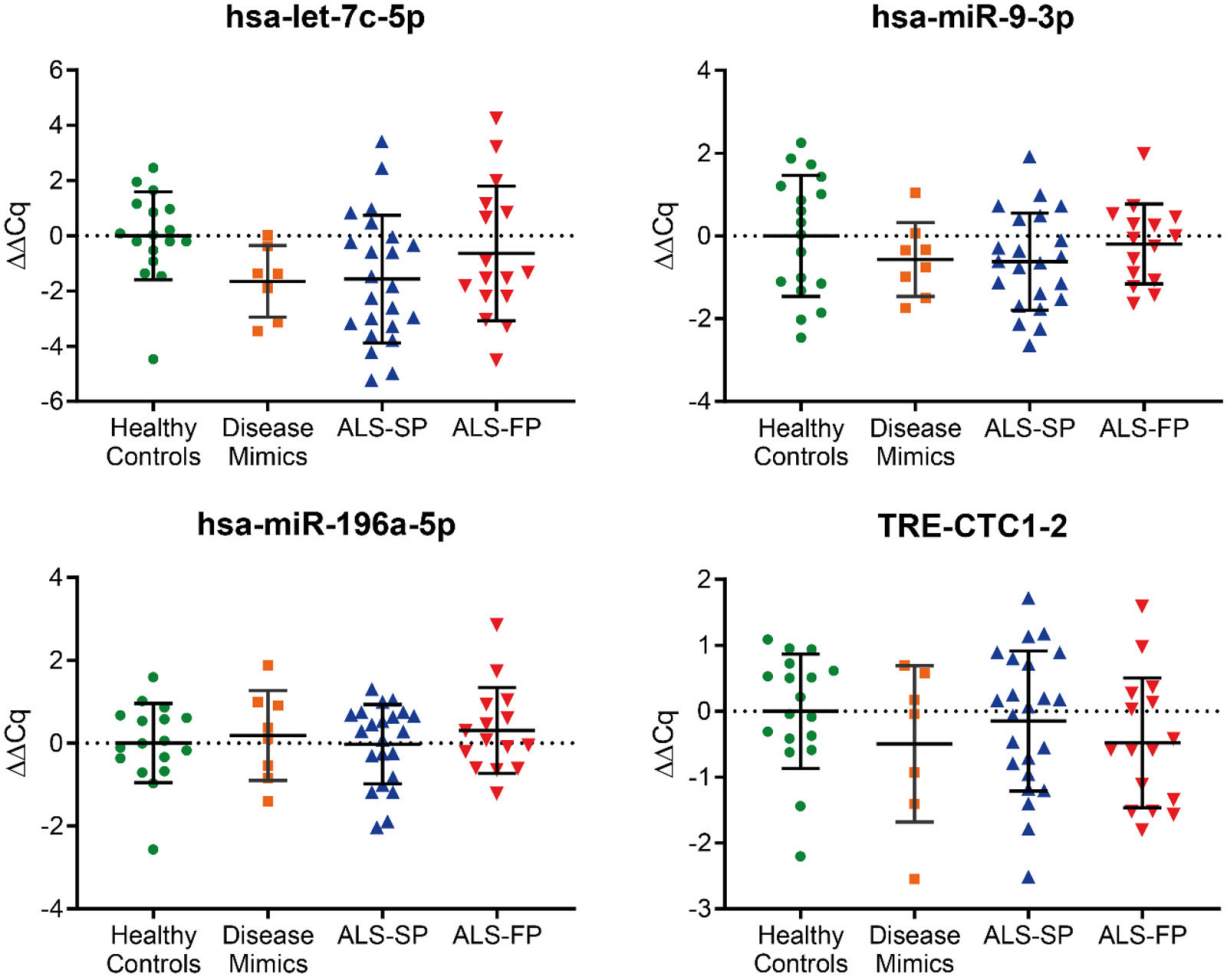
RT-qPCR profiling of ncRNA biomarker candidates in CSF from RNA-seq data. Those ncRNA transcripts that showed normal amplification during RT-qPCR across a majority of samples showed no significant dysregulation compared to the average of the healthy control samples. ALS-FP: fast-progressing ALS, ALS-SP: slow-progressing ALS; relative expression ± SD; normalised to geometric average of hsa-miR-9-5p and hsa-miR-501-3p; one-way ANOVA with Tukey’s.

For hsa-let-7c-5p, hsa-miR-9-3p, hsa-miR-196a-5p, and TRE-CTC1-2, we were able to show amplification across the majority of samples. However, there was no significant dysregulation of these targets identified between the groups, including the disease mimics which consisted of people with motor disorders similar to ALS (Figure 3). Grouping samples irrespective of progression rate continued to show no significant dysregulation (Supplementary Figure 1). Furthermore, the remaining miRNA showed little or no amplification with RT-qPCR in all or some samples, even with additional pre-amplification or cycling for particular ncRNA, suggesting that unlike serum, the expression of these ncRNA are not as ubiquitous across patient samples. Combined, these RT-qPCR data on individual samples did not confirm the RNA-seq analysis from pooled samples.

### Previously identified serum-based ncRNA biomarker candidates also not dysregulated in matched CSF samples

We have previously identified seven ncRNA that may be candidates as biomarkers for ALS in the serum [7], but the source of these biomarkers is unknown. We hypothesised that these changes could be reflective of changes in ncRNA expression in the motor system in the CNS. Such changes would therefore be reflected in changes in CSF since this biofluid is in close contact with the extracellular space of the brain and spinal cord and any consistent dysregulation in ncRNA expression between serum and CSF would suggest that the origin of these ncRNA are from the CNS.

**Figure 4.**
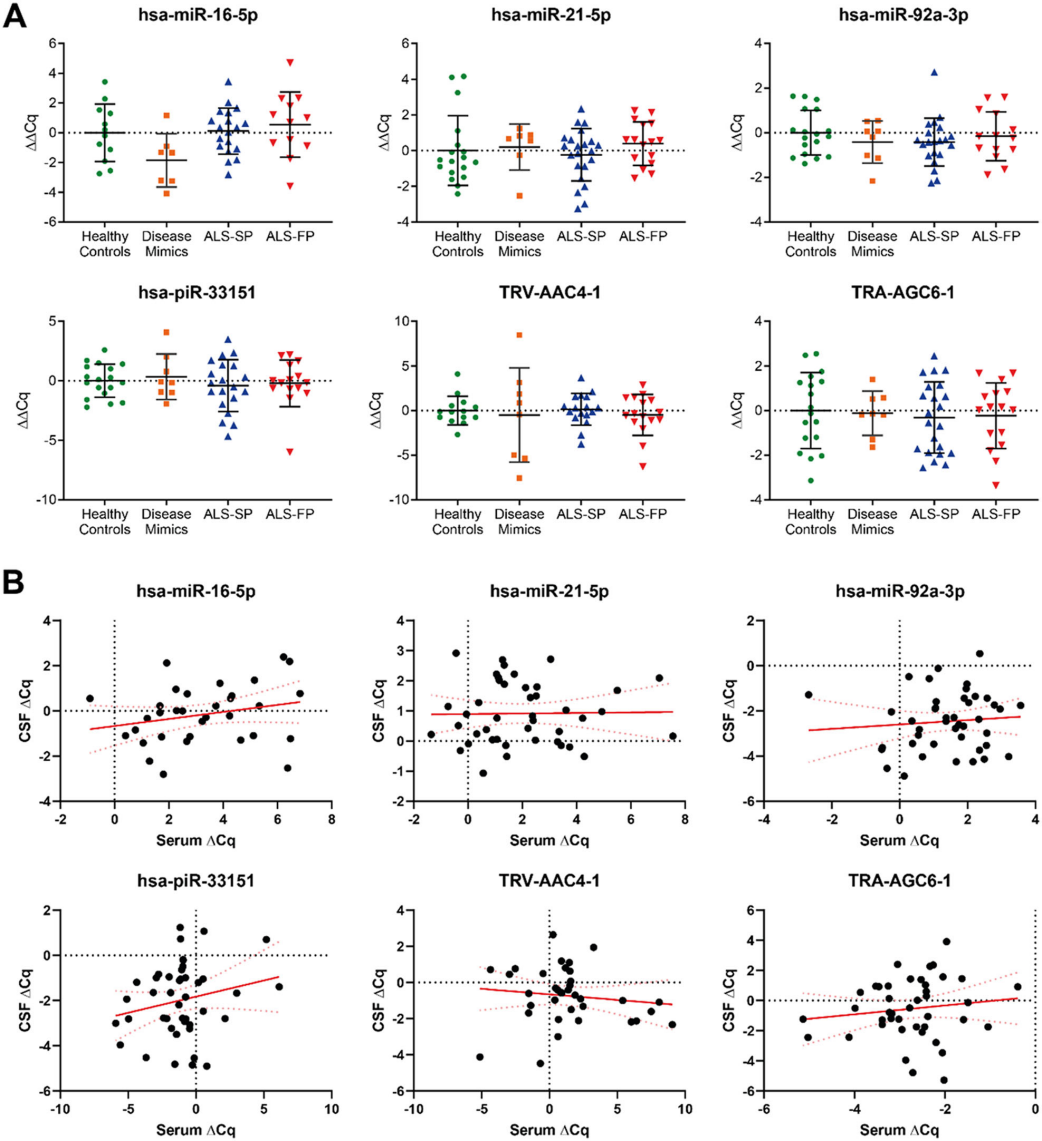
Expression of serum-based ncRNA biomarker candidates in CSF in the BioMOx cohort. (A) No significant dysregulation of serum-based ncRNA biomarker candidates from Joilin et al. [7] were detected in CSF compared to the healthy controls. (B) No significant correlation in expression between CSF and serum in matched patient samples in the BioMOx cohort across all groups. Data from serum samples were collected as part of the study in Joilin et al. [7]. ALS-FP: fast-progressing ALS, ALS-SP: slow-progressing ALS; relative expression ± SD; normalised to geometric average of hsa-miR-9-5p and hsa-miR-501-3p; relative expression: one-way ANOVA with Tukey’s; correlation: Pearson’s correlation.

Therefore, we profiled the expression of our seven ncRNA using RT-qPCR across our samples from healthy controls, disease mimics, and ALS patients (Figure 4(A)). Firstly, there was very limited detection of hsa-miR-206 in our CSF samples. Secondly, we found that unlike the serum, the remaining six ncRNA showed no significant differential expression across any of the three sample groups, and as above, grouping samples irrespective of progression rate continued to show no significant dysregulation (Supplementary Figure 2). Lastly, some of the CSF samples from the BioMOx cohort had matching serum samples from the same patient, so using previously collected data from our previous study [7], we undertook to see whether there was a correlative relationship between serum and CSF. However, this also showed no significant relationship across all the ncRNA biomarker candidates (Figure 4(B)). As such, this suggests that the CSF is most likely not the source of the ncRNA shown to be dysregulated in the serum of ALS patients and that these changes in ncRNA expression are not occurring globally in all tissues.

## Discussion

Using RNA-seq, we have observed that there are a range of ncRNA that are detectable in CSF and that there may be some dysregulation in their expression. However, they did not validate in RT-qPCR assays. As a result, we are unable to fully assess whether this dysregulation occurs in ALS patients and their potential utility as biomarkers for ALS. One reason for these difficulties may be due to the use of our pooling strategy. While pooling RNA from out samples together worked with serum in identifying biomarkers [7], this approach was not as successful with CSF based on the lack of validation with RT-qPCR. The aim of pooling our samples together was to help increase the signal to noise ratio as variable ncRNA expression will offset each other while commonly dysregulated ncRNA will be highlighted. However, while our RNA-seq analysis identified dysregulated ncRNA, RT-qPCR failed to confirm the dysregulation of these biomarker candidates. We suspect that this lack of confirmation was due to the low amounts of RNA present in CSF. Indeed, some transcripts showed no expression in some samples, which is an issue as any ideal biomarker should be detected in all samples robustly. However, by pooling the CSF samples, this may have caused the RNA content of some samples to obscure the lack of particular transcripts in other samples, artificially decreasing the expression in a pool and generate a false impression of dysregulation. This low amount of RNA and the resulting difficulty in profiling ncRNA in CSF, similarly encountered in another study [15], is likely due to the reduced number of tissues that can secrete molecules into the CSF in comparison to serum. As such, profiling ncRNA using RNA-seq from CSF may provide skewed results with pooling further obscuring this.

These observations suggest that for diagnostic biomarker profiling, serum may be more informative than CSF. It appears that ncRNA are lowly expressed in CSF and considering that it is less routinely obtained, the value in undertaking routine profiling of this biofluid for objective monitoring of the disease seems small. In contrast, serum collection from blood is more easily obtainable and routine, has abundant expression of ncRNA, and as described above could still reflect pathological processes such as ncRNA release from dying muscle. As such, from a clinical perspective, identifying ncRNA biomarkers in serum is the ideal proposition. However, identification of ncRNA in CSF could be done from unpooled samples if required, such as clinical trials affecting ncRNA, and if this provided validated targets may provide insight into pathology.

We also observed that there was no correlation between the ncRNA we identified as dysregulated in serum with that of CSF. This suggests that dysregulation in the serum is not underpinned by the CSF and that the source of these ncRNA is not from the CNS, though this does not discount that ncRNA are not dysregulated in cells within the CNS in ALS patients, contributing to the pathology to the disease. Therefore, other tissues are more likely to be the source of the ncRNA present in the serum. A chief candidate for this includes muscle tissue, which wastes away during the disease and is likely to lead to the cellular contents being released into the extracellular space and taken up into the blood. This is supported by the up-regulation of hsa-miR-206, which is a muscle enriched miRNA linked to ALS and shown to be important for the maintenance of the neuromuscular junction [20], in the blood of ALS patients [6–9], and has been shown to be up-regulated in blood in other diseases involving muscle atrophy [21,22]. Additionally, ncRNA such as miRNA and tRNA halves are known to act as paracrine signallers between cells and cause changes in gene expression and cellular function. This includes in ALS between astrocytes and neurons [23], and in other contexts between other cells, including peripheral immune cells [24–27]. As such, profiling of ncRNA in the CSF of ALS patients may not have been useful for biomarker discovery, but these data have provided insight into the potential source of the dysregulated ncRNA observed in serum and suggest other tissues and cells may be of greater interest.

## Supplementary Material

Supplemental Material

Supplemental Material

## Data Availability

The data that support the findings of this study are available from the corresponding author, MH, upon reasonable request.
