# Supplementary material for: Profiling non-coding RNA expression in cerebrospinal fluid of amyotrophic lateral sclerosis patients
Source: Ann Med. 2022 Oct 31;54(1):3069–78. doi: 10.1080/07853890.2022.2138530 (PMC9629092; doi:10.1080/07853890.2022.2138530)
Supplement: Supplemental Material [file IANN_A_2138530_SM0686.docx]

**
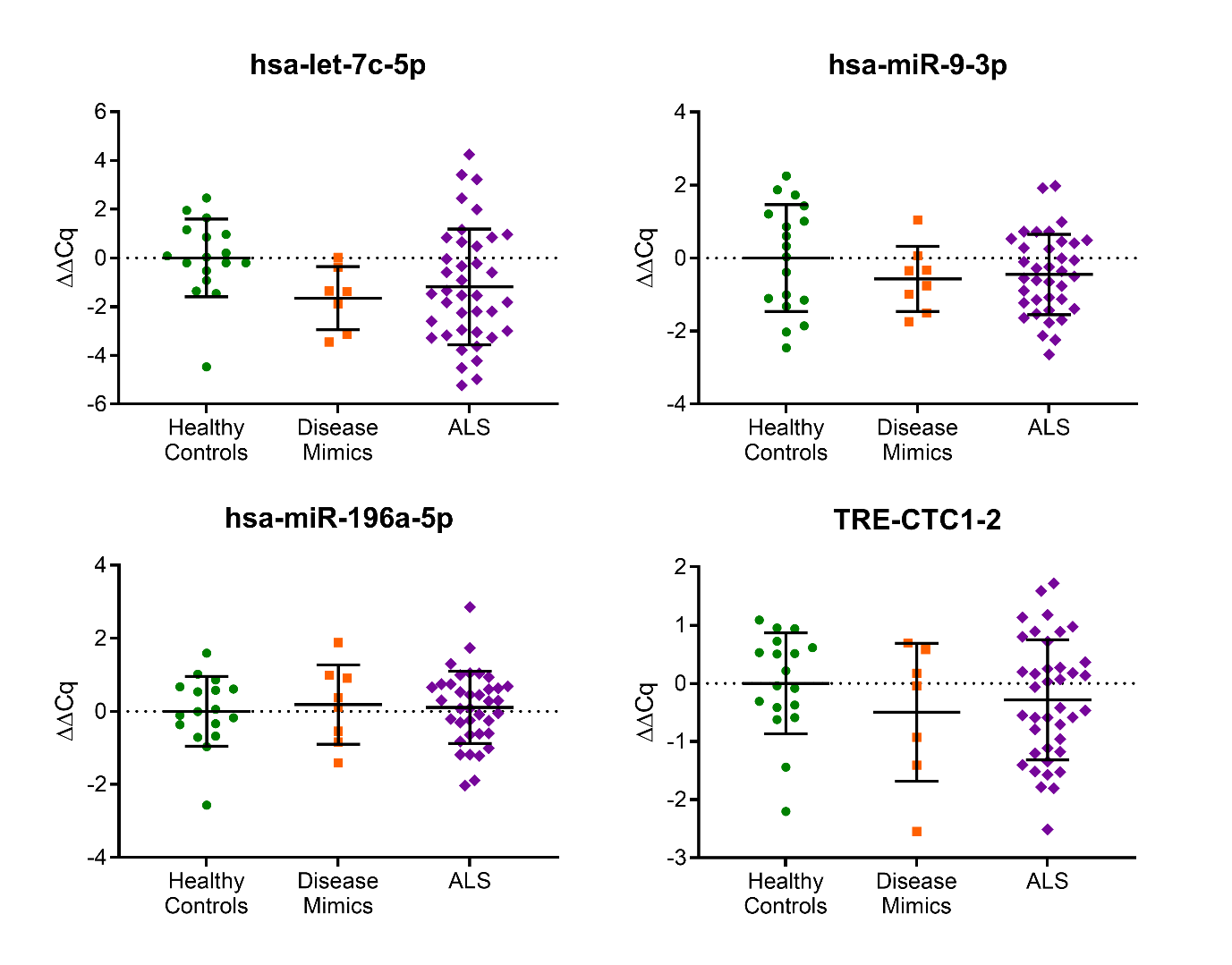
Supplementary Figure 1:** RT-qPCR profiling of ncRNA biomarker candidates in CSF from RNA-seq data with all ALS cases combined. Those ncRNA transcripts that showed normal amplification during RT-qPCR across a majority of samples showed no significant dysregulation compared to the average of the healthy control samples. Relative expression ± SD; normalised to geometric average of hsa-miR-9-5p and hsa-miR-501-3p; one-way ANOVA with Tukey’s.

**
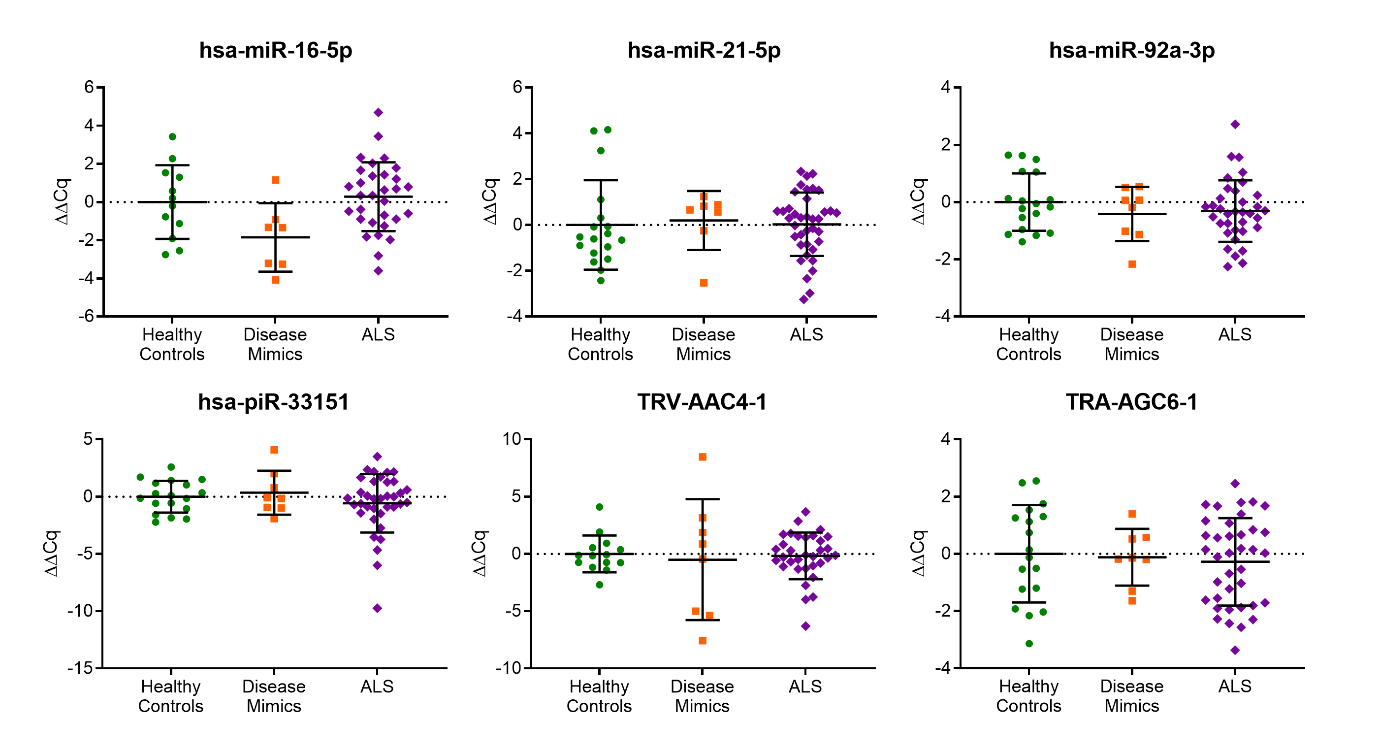
Supplementary Figure 2:** Expression of serum-based ncRNA biomarker candidates in CSF in the BioMOx cohort with all ALS cases combined. No significant dysregulation of serum-based ncRNA biomarker candidates from Joilin et al. (2020) were detected in CSF compared to the healthy controls. Relative expression ± SD; normalised to geometric average of hsa-miR-9-5p and hsa-miR-501-3p; relative expression: one-way ANOVA with Tukey’s.
